# Treatment effects of two pharmaceutical skin care creams for xerotic feet among persons with diabetes: Rationale and design of a two-armed double blind randomized controlled trial

**DOI:** 10.1016/j.conctc.2024.101372

**Published:** 2024-09-16

**Authors:** Anna Ericsson, Karin Borgström, Christine Kumlien, Magdalena Gershater Annersten, Tautgirdas Ruzgas, Johan Engblom, Petri Gudmundsson, Victoria Lazer, Skaidre Jankovskaja, Eva Lavant, Sophia Ågren-Witteschus, Sebastian Björklund, Saman Salim, Mikael Åström, Stefan Acosta

**Affiliations:** a Department of Care Science, Faculty of Health and Society, Malmö University, Malmö, Sweden; b Department of Biomedical Science, Faculty of Health and Society, Malmö University, Malmö, Sweden; c Biofilms – Research Center for Biointerfaces, Malmö University, Malmö, Sweden; d Department of Cardiothoracic and Vascular Surgery, Skåne University Hospital, Malmö, Sweden; e Department of Dermatology and Allergy Centre, Odense University Hospital, Odense, Denmark; f Department of Clinical Sciences, Lund University, Malmö, Sweden; g StatCons, Sweden

**Keywords:** Diabetes mellitus, Dry feet, Prevention, Foot-xerosis, Self-care

## Abstract

**Introduction:**

To minimize the risk of developing foot-ulcers, persons with diabetes are given the advice to daily inspect their feet and to apply skincare formulations. However, commercially available skincare products have rarely been developed and evaluated for diabetes foot care specifically. The primary aim of this randomized controlled trial (RCT) is to evaluate the effects in reducing foot xerosis in persons with diabetes without foot-ulcers using two skincare creams containing different humectants (interventions) against a cream base non-humectant (comparator). Secondary outcomes are to evaluate differences on skin barrier integrity, low-molecular weight biomarkers and skin microbiota, microcirculation including transcutaneous oxygen pressure, degree of neuropathy, and HbA1c between intervention-comparator creams.

**Methods:**

Two-armed double-blind RCT, registered in ClinicalTrials.gov Identifier: NCT06427889. With 80 % power, two-tailed significance of 2.5 % in each arm, 39 study persons is needed in each arm, total 78 persons, 98 including dropouts, to be able to prove a reduction of at least one category in the Xerosis Severity Scale with the intervention creams compared to the comparator. In one arm, each participant will treat one foot with one of the intervention creams (Oviderm® or Canoderm®), while the opposite foot will be treated with the comparator cream (Decubal®lipid cream), twice a day. If needed, participants are enrolled after a wash-out period of two weeks. The participants will undergo examinations at baseline, day 14 and day 28.

**Discussion:**

This RCT evaluate the potential effects of humectants in skin creams against foot xerosis in persons with diabetes.

## 1 Introduction

Diabetes mellitus is expanding globally, accounting for 2–5 % of the population in Europe. According to the International Diabetes Federation the prevalence of diabetes in Swedish adults is 6.8 % [1]. The prevalence of diabetic foot ulcers (DFU) is in the range of 4–10 % among the persons having diabetes [2] and the lifelong risk to develop a foot ulcer is as high as 25 % [3]. Approximately 50 % of people with diabetes and a foot ulcer have peripheral artery disease (PAD) and the presence of PAD significantly increases the risk of adverse limb and cardiovascular events [4]. Peripheral neuropathy alone, or in combination with PAD [5], is a serious complication to diabetes and can cause both dry skin and calluses leading to increased skin cracks and risk for development of DFU. DFU is a devastating complication that rapidly can lead to deep foot infection, amputation, sepsis, and death [4]. More than 60 % of all non-traumatic lower extremity amputations are due to diabetes mellitus [6]. DFU management is very resource demanding with great impact on costs for health care systems. In the event of amputation, the costs for rehabilitation are significant [7]. Life expectancy is reduced for persons with DFU [8,9].

In addition, patients with DFU report pain, mobility limitation, discomfort feelings and impairment of quality of life [10,11]. Hence, it of outmost importance to prevent the development of DFU [12]. To minimize the risk to develop ulcers, patients with diabetes are given the recommendation from health care professionals to daily inspect their feet and to use emollient [4]. In brief, an emollient is normally referred to a skincare product that softens and hydrates the skin by forming a protective barrier to reduce transdermal water loss. A moisturizer, on the other hand, is a formulation designed to hydrate the skin, usually by incorporating small polar substances referred to as humectants, which have water attracting properties. Thus, both emollients and moisturizers aim at promoting skin hydration but their mode of action differs. However, commercially available skincare products for treating dry skin in individuals with diabetes are seldom designed specifically for this demographic's skincare needs. Nevertheless, Garrigue et al. [13] showed that application of Pedimed® cream (contains humectants, i.e., 10 % glycerine, 5 % urea and 1 % lactic acid) twice a day, significantly reduced foot xerosis over its vehicle after 2 and 4 weeks of treatment in patients with diabetes [13]. In another study [14], the efficacy of improving skin dryness of the dorsal and plantar regions of feet of patients with type 2 diabetes was investigated by comparing Ureadin® (containing 5 % urea) with Dexeryl® (containing 15 % glycerol). Following visual inspection, it was concluded that application of Ureadin® resulted in significantly greater skin hydration compared to Dexeryl® with an 89 % reduction in dry skin area severity index score in comparison with baseline values [14].

Despite a few studies showing reduction of xerosis of diabetic skin by application of skincare products, there are no firm recommendations of skincare routines for persons with diabetes, specifying which type of creams they should use to reduce the risk of ulcers.

Therefore, the primary objective of this study is to evaluate the reducing effect of skin xerosis using two pharmaceutical skin care creams (cream A and cream B) containing different humectants, compared to a cream without any humectant (cream C).

## 2 Materials and methods

*Overall-Design*: Two-armed double-blind randomized controlled clinical trial (RCT).

### 2.1 Objectives of the study

The primary objective of this RCT is to evaluate the effect of two, humectant-containing pharmaceutical skin creams in reducing skin xerosis on feet of persons with diabetes without foot ulcers. The results will be compared to skin treatment with a humectant-free cream.

### 2.2 Participants, setting, recruitment and randomization

Persons above 18 years with diagnosed diabetes having dry feet without foot ulcers will be contacted by the researchers and study nurses through diabetes societies and social media for the opportunity to be included in the study. This interventional study will be conducted within the facilities of Malmö University, Sweden.

#### 2.2.1 Inclusion criteria

• Persons above 18 years of age diagnosed with any type of diabetes mellitus, with self-experienced dry skin.
• Dry skin on both feet with a Xerosis Severity Scale (XSS) [15] score between two to six. No more than one XSS score in difference between the subjects' feet.
• Ability to understand sufficient Swedish language to complete the necessary forms and understand the procedure of the study.

#### 2.2.2 Exclusion criteria

• Known sensibility to any of the ingredients in the products.
• Other diagnosed skin disease on the feet.
• Active lesions on either foot.
• Treatment with local medications, including topical moisturizers or keratolytic agents on the feet, within two weeks before the beginning of the study.
• Potential study subjects judged unable to comply with treatment schedule and study specific information.
• Female of childbearing potential that do not use effective medically accepted contraception.

#### 2.2.3 Enrollment and randomization

Persons with diabetes will be introduced about the study at society meetings and at social media. Potential study participants that are suitable (according to the study criteria) for the study will be contacted and informed in more details about the study by the research nurses. Furthermore, a welcome letter will be sent by post to the participants with practical information. If acceptance and pass of criteria, informed consent form will be signed and the potential participants will be recorded in the research software program Research Electronic Data Capture® (REDCap) version 13.1.37. The participants will then be randomized and will adhere to the clinical study schedule and specific study procedures. The enrolled study participants will be given an autonomic code (in REDCap) as soon as the participants have signed the consent form. According to the code the participants will be randomized into different treatment, unknown which, for both the participants and the investigators (double-blind). Apart from the treatment randomization the participants will be randomized to which foot that will be treated with which cream. One foot will be treated with one interventional cream (cream A or B) and the contralateral foot with the comparator humectant-free cream (C).

#### 2.2.4 Sample size estimation

This is a superiority RCT, where either of the intervention creams is hypothesized to be superior to the comparator cream in reducing XSS score during a four-week application period. To be able to prove this hypothesis, a total of 98 participants, 39 persons with diabetes in each arm for analysis, is needed, which is based on the following: A change in XSS score ≤1 is not regarded clinically important. The estimated intra-individual standard deviation for the difference in XSS score is 2.0. With 80 % power, two-tailed significance of 2.5 % in every arm, 39 evaluable study persons is needed in every arm, a total of 78 persons. If 20 % of persons are estimated not to be fully evaluable, sample size increases to 98 (78/0.8).

The following formula will be used to calculate the number of patients needed in each part of the trial [15,16] (N = number of fully evaluable patients in each part of the trial):

$$N=\frac{\sigma_{d}^{2}\times {\left(\lambda_{\alpha }+\lambda_{\beta }\right)}^{2}}{\Delta^{2}}$$

In the formula above σ_d_ is the standard deviation for the difference between the change in each treatment, Δ is the difference to detect between the treatments (a difference of at least 1 is assumed to be clinically relevant), λ_x_ is the x:th percentile of the standardized normal distribution. As the test is two-sided and the significance level should be 0.0250 (in order to keep the family-wise type I error at 5 % and there are two comparisons: Canoderm® versus Decubal® and Oviderm® versus Decubal) then λ_α_ = 2.1414. If the power is 80 % then λ_β_ = 0.8416 and if the power is 90 % then λ_β_ = 1.2816.

#### 2.2.5 The creams

The intervention creams are Oviderm (Oviderm 25 % cream, Galenica AB, Sweden) and Canoderm (Canoderm 5 % cream, ACO HUD Nordic AB, Sweden), are used as moisturizing treatment for dry skin. Oviderm contains propylene glycol at a concentration of 25 %, while Canoderm contains 5 % propylene glycol and 5 % urea. Both propylene glycol and urea are low molecular weight substances with water attracting properties and therefore normally described as humectants with good capacity to improve skin hydration [17]. In addition, urea is keratolytic and used at high concentrations for the treatment of hyperkeratotic skin disorders [17]. Propylene glycol on the other hand, is widely used in pharmaceutical formulations as a solvent, or vehicle, for substances with low solubility in water and also demonstrated to have penetration enhancing properties [18]. Propylene glycol in this concentration (25%) has additionally some antimicrobial properties [19]. Studies have also shown that urea and propylene glycol enhances the molecular mobility skin barrier lipids and keratin filaments at relatively dry conditions [20,21]. Notably, this effect is not solely due to increased water content of the skin but rather appears to be related to preserved fluidity of the skin barrier molecular components under dehydrating conditions [20]. Urea is also known for its skin barrier strengthening effect [22]. The comparator, Decubal lipid cream (Decubal Lipid Cream, Karo Healthcare AB, Sweden), does not contain humectants. Nonetheless, it effectively forms a semi-occlusive barrier on the skin, minimizing water loss through vaporization and thereby enhancing hydration. This effect should be common to all three creams employed.

#### 2.2.6 Package, labelling and storage

##### 2.2.6.1 Package

All three creams will be filled into similar pump containers to ensure the blinding process. The filled containers will be packed as kits. Each kit will contain two containers, with two different creams, either Oviderm together with Decubal lipid cream or Canoderm together with Decubal lipid cream. One of the kit type will be given to each participant after the randomization procedure.

##### 2.2.6.2 Labelling

The filled cream containers will be labelled as well as the card box kit package. This will be done in accordance with the Swedish Medicines Agency collection LVFS 2011:19 (https://lagen.nu/lvfs/2011:19), §7, page 15. The container in the kit will be marked with the foot to be treated (right or left).

##### 2.2.6.3 Storage

The study creams will be stored at the Contract Research Organisation (CRO), Bioglan AB Malmo, Sweden and the research team will order the products when needed. The study creams should be kept in a room with maximum temperature of 25 °C. The study participants will be informed about this.

### 2.3 Intervention

If the participants are using any topical products (i.e. oils, ointments, creams or gels) a wash-out period of two weeks is needed. All participants will be assessed against inclusion-exclusion criteria and if eligible they will be asked in the welcome letter to discontinue treatment for two weeks and to return to the study clinic. The welcome letter will inform them about the address and time and date for the appointments. If they have not used any foot cream for the last two weeks and are judged eligible, they will be assessed for a number of parameters including skin barrier characteristics, criteria, and randomized already at the first visit. The participants will receive a kit with two identical containers, marked “left foot” and “right foot”. The participants will be informed about the importance to use the same marked container for the same treatment area during the whole study period. After application on one foot, the participants need to wash their hands, which they also will be informed about. The study creams (described below) will be applied on the feet twice a day. Each participant will be asked to mark the calendar each morning and evening when having applied cream in an individual schedule, which will be distributed at the start of the study.

The efficacy (the difference of change in xerosis between baseline until 4 weeks post baseline between intervention and comparator groups), and safety, between Oviderm cream against Decubal lipid cream in one arm, and Canoderm cream against Decubal lipid cream in the other arm, will be evaluated. The study trial period of four weeks was chosen based on shown effects in the intervention arm in previous RCTs [13,14] on foot xerosis in persons with diabetes mellitus.

#### 2.3.1 Outcomes

##### 2.3.1.1 Primary outcomes

The primary outcome in this study, which the power calculations are based on, is the change in xerosis (dry skin), after four-week application of creams containing humectant compared to humectant-free cream on the diabetic feet. XSS score will be assessed and evaluated at baseline, i.e., visit 1 or 2 (if washout period needed), visit 2/3 (after two weeks after baseline-week application) and visit 3/4 (four weeks after baseline). Assessment will be performed using the 7-graded (from 0 to 6) XSS score. The classification of the scale is described in Table 1. Foot xerosis severity will be determined based on the most severe located dryness of the foot, and this location will be documented in REDCap.

**Table 1:** The xerosis severity scale.

Classification	Description	Category
Mild	Normal Skin	0
	Dusty appearance, occasional minute skin flakes	1
	Generalized dusty appearance, many minutes skin flakes	2
Moderate	Defined scaling with flat borders	3
	Well defined heavy scaling with raised borders, shallow fissures	4
Severe	Large scale plates, fissures	5
	Large scale plates, deep erythematous fissures	6

Digital photographs with Canon EOS (Electro-Optical System) 750D, Canon Inc, Tokyo, Japan of the participants' feet will be taken for documentation in REDCap to be objectively evaluated by a dermatologist, and for XSS validation, by a second dermatologist. The Canon EOS 750D is capable of producing great photos and has been updated with a 24.2 megapixel sensor and built in Wi-Fi making it an excellent entry level DSLR (digital single-lens reflex camera). In a report published in 2018, a DSLR camera was successfully used to visually classify heel xerosis grade using similar xerosis assessment scale as outlined in the present study protocol, and to evaluate the effects of moisturizer on heel skin dryness [23]. The dermatologists will not have any personal contact with the study participants and will be blinded to cream exposure to evaluate dryness of the feet.

##### 2.3.1.2 Secondary outcomes

The secondary outcomes are supporting evidence of differences in efficacy of creams on skin barrier integrity, and changes in health-related quality of life during the study period. Skin barrier integrity and hydration will be obtained by non-invasive biophysical measurements as follows.

• Trans epidermal water loss (TEWL) is a key indicator of the barrier function of the stratum corneum (SC). Elevated TEWL typically indicates a compromised skin barrier, while lower TEWL is associated with good SC barrier properties and healthy skin [24].
• Skin hydration by measurement of skin impedance (skin resistance and skin capacitance). It is well-known that maintaining healthy skin relies heavily on adequate skin hydration [25]. Standard methods for assessing skin hydration involve measuring the skin's electrical properties, which vary with the skin's water content. In this work, the skin hydration will be assessed by measuring the resistance and capacitance by electrical impedance spectroscopy [26]. Increased skin capacitance (increased dielectric constant of skin) indicates higher skin hydration; thus healthier skin barrier [26].
• Skin surface pH provides an indication of host-microbiota interaction. Acidic pH value (*e.g.*, pH 5.5) is usually related to the dominance of commensal bacteria on the skin. Shifting skin surface pH towards basic values (*e.g.*, pH 6.8) might indicate an increased inhabitance of skin by pathogenic microbiota [27].
• Health related quality of life assessed with EuroQoL 5 level (EQ-5D-5L) instrument.

To support the outcomes by molecular characteristics of skin and physiological health parameters relevant to diabetes the following assessments will be made.

• Macrocirculation measured as ankle-brachial index and toe-brachial index. Microcirculation including transcutaneous oxygen pressure (Laser doppler/transcutaneous O_2_ -electrode/laser doppler flow with heat provocation) (Peripheral circulation is measured by Periflux 6000 combined; Perimed AB, Stockholm, Sweden)
• Degree of neuropathy (multi-frequency vibrametry) (VibroSense Meter; VSM II, Vibrosense Dynamics AB, Malmö, Sweden). Assessment of vibration perception threshold at 125 Hz with multi-frequency vibrometry has shown to have a strong correlation with nerve conduction studies in diabetic peripheral neuropathy [28].
• Molecular biomarkers including microbiota will be analyzed after a non-invasive sampling from the surface of the skin.
• HbA1c by using a stick test for collecting capillary blood in the middle finger.
• XSS scores are determined by study investigators at study inclusion and will be validated by a dermatologist. Photographs of the feet will be taken to validate XSS grading and for documentation of the colour and surface structure of the skin.

### 2.4 Data management

For the current study data will be entered into and monitored in REDCap, version 13.1.37. The study is divided into four phases: *Screening, Initial evaluation and randomization, Treatment and Final evaluation.* The collection of data will run over a period of four weeks. Data that will be collected during the screening, visit 1 at the study clinic, will be year of birth, gender, specification of diabetes type, year of diabetes diagnosis, medical history, medication, date of study entry and concomitant medication. Health declaration will be collected and informed consent will be signed. All participants will be asked on forehand (Welcome letter and in the phone call) to have a washout period of 14 days before the first visit at the study clinic. If the participants have not used creams (have had their wash-out period) for two-weeks, visit one and visit two will be merged and baseline data will be collected (see below for descriptions). If the participants need to come back after the washout, that will be the second visit for them. Baseline data will include; XSS with photos, TEWL, hydration, skin pH, Vital signs, circulation tests (both micro and macro), neuropathy, collection of low molecular biomarker and microbiota from the surface of the skin, HbA1c, Health-related Quality of Life (EQ-5D, the Swedish tested version, with approved registration number 46933), adverse events, product accountability. Seven days after the baseline the participants will be contacted over phone by the research nurses. Approximately 14 days (12–16 days) after baseline data is collected, XSS with photos, TEWL, hydration and skin pH will be taken.

At the last visit 26–30 days after the study start, the same tests will be conducted as during visit 1 (Table 2).

**Table 2:** Flowchart of the studyplan and collection of the data.

Events	Visit 1 Screening & Baseline (if washout period is not needed) 120 min	Visit 2 Baseline (if wash-out period is needed) Day 0 100 min	Phone call Day 7 (5–9) 30 min	Visit 3 Day 14 (12–16) 60 min	Visit 4 Day 28 26–30 120 min
Signing Consent form	x				
Allocation of study number	x				
Eligibility criteria	x				
Demographic data	x				
Medical history of diabetes	x				
Comorbidity (Health declaration)	x				
Concomitant medication	x				
Extra eligibility test if 2-weeks wash-out	x	x			
Neuropathy	x	x			x
Macro-and Micro circulation	x	x			x
Vital signs	x	x			
Dispensing of study product kit	x				
Weight of cream container	x	x		x	x
XSS including photography of the foot	x	x		x	x
TEWL	x	x		x	x
Skin hydration/Impedance	x	x		x	x
Skin pH	x	x		x	x
Molecular biomarkers sampling from skin	x	x			x
Microbiota sampling from skin	x	x			x
Analysis of HbA1c by prick test	x	x			x
Adverse Events	x	x	x	x	x
EQ-5D-5L	x	x			x
Product accountability	x	x	x	x	x

Visits at the study clinic will be scheduled by the study investigators together with the study participants, according to a time schedule. The participants will be asked about any adverse events during all visits and over the phone call.

#### 2.4.1 Data management

The principal investigator (PI) [12] will have the main responsibility for the monitoring of the data but data will be registered by the research study investigators. Furthermore, the PI will ensure the design of the trial and will have overseen for data integrity and data security.

### 2.5 Statistical analysis

The primary analysis principle applied will be the intention-to-treat. All statistical analysis and data management will be performed using SPSS for Windows, version 28.0 (IBM, Armonk, NY, USA). Measurements, such as XSS score, TEWL, pH, impedance, abundance of the biomarkers will be used as continuous variables and treatment with the creams, gender, and patients grouping parameters will be used as categorical variables. Descriptive statistics such as number of participants, mean, median, standard deviation, minimum and maximum values will be summarized and tabulated. XSS score will be independently evaluated by a random sample of twenty photographs by a second dermatologist, for validation purposes, to be able to assess inter-rater reliability by calculating intraclass correlation with 95 % confidence intervals. The primary outcome of the study is to compare the change in XSS score from baseline until the end (4 weeks) due to treatment with moisturizing creams vs a comparator product. Prior to analysis of the XSS differences between the treatment groups, the distribution of the data will be tested for normality by performing Shapiro-Wilk and Kolmogorov-Smirnov tests, as well as inspecting quantile-quantile (QQ) plots. Homogeneity of variances will be tested by performing Levene's test. Comparison between two related groups, *e.g.*, application of the formulation on the left and right foot, will be done by paired two-sample *t*-test, whereas for unrelated two groups two-sample *t*-test will be performed. When neither homogeneity of variances nor assumption of normality will be fulfilled, a non-parametric Wilcoxon rank sum test will be applied. In case of comparison between more than two groups, *e.g.*, the effect of three creams, one-way ANOVA with multiple comparisons (Tukey's test) will be used. Alternatively, if assumptions of normality or homogeneity of variances will not be fulfilled, non-parametric Kruskal Wallis test will be performed. The effect of covariables, *e.g.*, age, on main effect, *e.g.*, XSS change upon the treatment with the cream, will be tested by performing ANCOVA analysis. To seek for the measurements capable to explain and predict the effectiveness of the diabetic foot treatment with different creams more complex statistical analysis will be performed. Principal component analysis (PCA), multiway ANOVA and mixed models, and multiple regression analysis and model selection will be performed on the data collected. Level of statistical significance was set to p<0.05.

### 2.6 Reporting

We will report according to the CONSORT 2010 checklist [29] and CONSORT 2010 Explanation and Elaboration guidelines [30].

### 2.7 Ethical consideration

Signed informed consent forms will be obtained from all participants prior to the study start. The study has been approved by the Swedish Medical Products Agency, application number 2022-500907-27-01-SM (substantial modification −1), date of approval 20231101. This RCT is registered the 23 of April 2024, at ClinicalTrials.gov (https://clinicaltrials.gov): Identifier: NCT06427889.

## 3 Discussion

The prevalence of diabetes mellitus is increasing in the world, 537 million adults suffer from this condition, and it is estimated to raise [1]. DFU is a devasting complication, ending up in major amputation and shorter life-expectancy for many. Prevention for DFU is therefore a global priority.

Better foot care with hydration may result in healthier feet but also to better vigilance of foot status in persons with diabetes. To be able to show differences in change in skin xerosis when using creams containing humectant compared with humectant-free cream on dry feet in persons with diabetes, might lead to specific recommendations for products that can be used for prevention of DFU. Furthermore, the results of this study can fill the gap of lack of documented effect when using different creams for persons with diabetes.

A potential limitation of this RCT could be that persons with diabetes accepting and attending this study is among those with most healthy feet in a population of diabetes, a well-known phenomenon in population-based screening studies [31]. Consequently, there is a risk that XSS scores at baseline are at the lower range, close to 2, where there is not much room for improvement by intense foot hydration with any of the creams. Determination of XSS scores is not always clear-cut and there may be discrepancies between raters. Therefore, we plan to perform an inter-rater reliability assessment, where twenty randomly selected photographed feet are selected and assessed by two independent dermatologists. The study protocol with at least three visits may for some participants be difficult to comply with, and there is a risk for attrition bias, where persons living far away from the centrally located research centre in Malmö, or elderly with higher degree of comorbidity, drop out from the study. The strengths are the design of two-armed double-blinded RCT, where the patients both feet are randomized, one receiving one of the intervention creams and the contralateral foot by default receiving the comparator cream. In this way, the participants feet act as a perfect matched pair of intervention-control, where patient-related factors are identical. Comprehensive evaluation of skin integrity barrier, microcirculation and neuropathy during the study period will hopefully result in advance in the understanding of disturbances of dry feet in persons with diabetes, and how to counteract.

## Funding

We acknowledge the 10.13039/100003077Knowledge Foundation (20190010), Region Skåne and Malmö University for financial support.

The Oviderm and Canoderm creams will be donated from the pharmaceutical companies Galenica, Malmö, Sweden and Perrigo Nordic, Kista, Stockholm, Sweden, respectively.

Disclose any financial and personal relationships. The authors have no personal or financial relationships with the companies providing the creams of this study.

## CRediT authorship contribution statement

**Anna Ericsson:** Writing – original draft. **Karin Borgström:** Validation. **Christine Kumlien:** Writing – original draft, Validation. **Magdalena Gershater Annersten:** Validation. **Tautgirdas Ruzgas:** Writing – review & editing, Writing – original draft, Validation, Formal analysis. **Johan Engblom:** Validation. **Petri Gudmundsson:** Validation. **Victoria Lazer:** Validation. **Skaidre Jankovskaja:** Validation, Formal analysis. **Eva Lavant:** Validation. **Sophia Ågren-Witteschus:** Validation. **Sebastian Björklund:** Validation. **Saman Salim:** Validation. **Mikael Åström:** Formal analysis. **Stefan Acosta:** Writing – review & editing, Writing – original draft.

## Declaration of competing interest

The authors declare that they have no known competing financial interests or personal relationships that could have appeared to influence the work reported in this paper.

## References

[1] Sun H., Saeedi P., Karuranga S., Pinkepank M., Ogurtsova K., Duncan B.B. (2022). IDF Diabetes Atlas: global, regional and country-level diabetes prevalence estimates for 2021 and projections for 2045. Diabetes Res. Clin. Pract..

[2] Fu X.L., Ding H., Miao W.W., Mao C.X., Zhan M.Q., Chen H.L. (2019). Global recurrence rates in diabetic foot ulcers: a systematic review and meta‐analysis. Diabetes/metabolism research and reviews.

[3] Boulton A.J., Whitehouse R.W. (2023).

[4] Fitridge R., Chuter V., Mills J., Hinchliffe R., Azuma N., Behrendt C.-A. (2023). The intersocietal IWGDF, ESVS, SVS guidelines on peripheral artery disease in people with diabetes mellitus and a foot ulcer. J. Vasc. Surg..

[5] Apelqvist J., Elgzyri T., Larsson J., Löndahl M., Nyberg P., Thörne J. (2011). Factors related to outcome of neuroischemic/ischemic foot ulcer in diabetic patients. J. Vasc. Surg..

[6] Norvell D., Thompson M., Boyko E., Landry G., Littman A., Henderson W. (2019). Mortality prediction following non-traumatic amputation of the lower extremity. Journal of British Surgery.

[7] Kurkela O., Nevalainen J., Arffman M., Lahtela J., Forma L. (2022). Foot-related diabetes complications: care pathways, patient profiles and costs. BMC Health Serv. Res..

[8] Armstrong D.G., Tan T.-W., Boulton A.J., Bus S.A. (2023). Diabetic foot ulcers: a review. JAMA.

[9] Heald A.H., Stedman M., Davies M., Livingston M., Alshames R., Lunt M. (2020). Estimating life years lost to diabetes: outcomes from analysis of National Diabetes Audit and Office of National Statistics data. Cardiovascular endocrinology & metabolism.

[10] Polikandrioti M., Vasilopoulos G., Koutelekos I., Panoutsopoulos G., Gerogianni G., Babatsikou F. (2020). Quality of life in diabetic foot ulcer: associated factors and the impact of anxiety/depression and adherence to self-care. Int. J. Low. Extrem. Wounds.

[11] Vileikyte L. (2008). Psychosocial and behavioral aspects of diabetic foot lesions. Curr. Diabetes Rep..

[12] Kumlien C., Acosta S., Björklund S., Lavant E., Lazer V., Engblom J. (2022). Research priorities to prevent and treat diabetic foot ulcers—a digital James Lind Alliance Priority Setting Partnership. Diabet. Med..

[13] Garrigue E., Martini J., Cousty-Pech F., Rouquier A., Degouy A. (2011). Evaluation of the moisturizer Pédimed® in the foot care of diabetic patients. Diabetes Metabol..

[14] Federici A., Federici G., Milani M. (2012). An urea, arginine and carnosine based cream (Ureadin Rx Db ISDIN) shows greater efficacy in the treatment of severe xerosis of the feet in type 2 diabetic patients in comparison with glycerol-based emollient cream. A randomized, assessor-blinded, controlled trial. BMC Dermatol..

[15] Jennings M.B., Logan L., Alfieri D.M., Ross C.F., Goodwin S., Lesczczynski C. (2002). A comparative study of lactic acid 10% and ammonium lactate 12% lotion in the treatment of foot xerosis. J. Am. Podiatr. Med. Assoc..

[16] Lachin J.M. (1981). Introduction to sample size determination and power analysis for clinical trials. Contr. Clin. Trials.

[17] Lodén M. (2003). Role of topical emollients and moisturizers in the treatment of dry skin barrier disorders. Am. J. Clin. Dermatol..

[18] Carrer V., Alonso C., Pont M., Zanuy M., Córdoba M., Espinosa S. (2020). Effect of propylene glycol on the skin penetration of drugs. Arch. Dermatol. Res..

[19] AB LS. https://www.fass.se/LIF/menydokument?userType=0&menyrubrikId=11094:FASS;[20240118.144022-8d2c20e6:[.

[20] Björklund S., Andersson J.M., Pham Q.D., Nowacka A., Topgaard D., Sparr E. (2014). Stratum corneum molecular mobility in the presence of natural moisturizers. Soft Matter.

[21] Kis N., Gunnarsson M., Berkó S., Sparr E. (2022). The effects of glycols on molecular mobility, structure, and permeability in stratum corneum. J. Contr. Release.

[22] Piquero-Casals J., Morgado-Carrasco D., Granger C., Trullàs C., Jesús-Silva A., Krutmann J. (2021). Urea in dermatology: a review of its emollient, moisturizing, keratolytic, skin barrier enhancing and antimicrobial properties. Dermatology and therapy.

[23] Choi J., Kim E., Jang S., Kim A., Lee T., Lee H. (2018). A new technique for evaluating heel xerosis grade and the effects of moisturizer on heel skin dryness. Skin Res. Technol..

[24] Akdeniz M., Gabriel S., Lichterfeld‐Kottner A., Blume‐Peytavi U., Kottner J. (2018). Transepidermal water loss in healthy adults: a systematic review and meta‐analysis update. Br. J. Dermatol..

[25] Verdier‐Sévrain S., Bonté F. (2007). Skin hydration: a review on its molecular mechanisms. J. Cosmet. Dermatol..

[26] Morin M., Ruzgas T., Svedenhag P., Anderson C.D., Ollmar S., Engblom J. (2020). Skin hydration dynamics investigated by electrical impedance techniques in vivo and in vitro. Sci. Rep..

[27] Fukuda K., Ito Y., Furuichi Y., Matsui T., Horikawa H., Miyano T. (2024). Three stepwise pH progressions in stratum corneum for homeostatic maintenance of the skin. Nat. Commun..

[28] Ekman L., Dahlin L.B., Andersson G.S., Lindholm E. (2024). Diagnostic contribution of multi-frequency vibrometry to detection of peripheral neuropathy in type 1 diabetes mellitus compared with nerve conduction studies. PLoS One.

[29] Schulz K.F., Altman D.G., Moher D., the C.G. (2010). CONSORT 2010 Statement: updated guidelines for reporting parallel group randomised trials. BMC Med..

[30] Moher D., Hopewell S., Schulz K.F., Montori V., Gøtzsche P.C., Devereaux P.J. (2012). CONSORT 2010 explanation and elaboration: updated guidelines for reporting parallel group randomised trials. Int. J. Surg..

[31] Manjer J., Carlsson S., Elmståhl S., Mattisson I., Berglund G., Gullberg B. (2001). The Malmö Diet and Cancer Study: representativity, cancer incidence and mortality in participants and non-participants. Eur. J. Cancer Prev..

